# Notes and News

**Published:** 1894-12-29

**Authors:** 


### Notes and News.

Collected and Communicated.

A new children's and general hospital has been opened at Leyton.

The Order of the Hospital of St. John of Jerusalem has lost their Chancellor through the sudden death of Sir Edmund Lechmere, M.P.

President Cleveland earnestly recommends the formation of a National Board of Health for the United States.

So seriously is the question of the contagiousness of phthisis regarded in Canada that at Toronto the Board of Education has decided to forbid children suffering from the disease to attend public schools.

The University of Utrecht has succeeded in retaining the services of Professor von Eiselberg, who was offered succession to Professor Gussenhauer at Prague. Professor von Eiselberg will receive not only an increased emolument, but a sum of 1,500 florins also for the organisation of a surgical department of the University of Utrecht. At Vienna Professor Isidor Schnabe, of Prague, will occupy the vacant chair of ophthalmology.

The use of the nomenclature of diseases used by the English Royal College of Physicians and Surgeons has been abolished by the United States medical department of the navy and a new classification substituted. This classification, it is stated, will be appreciated by the entire profession, being based on modern requirements. The new system, which is to begin with the new year, will, it is hoped, be the means of securing valuable and accurate statistics.

Dr. Salzmann, of Essling, in Germany, has been collecting statistics on the average duration of the physician's life. As with the general community, the duration of life is longer at the present time amongst doctors than in the past. In the sixteenth century the average was 36·5 years; now it amounts to 56·7. Improved sanitation and a system of antiseptics acts no less in favour of prolonged life amongst the profession than in the case of their patients and the public generally.

The St. Pancras Infirmary, situated close to Waterlow Park, is having a most valuable addition to its buildings, in the form of a proper nurses' home. It is many years since the Nightingale system, under a trained matron, was inaugurated at this institution, and the want of a nurses' home has been seriously felt by the good workers who have devoted themselves so unweariedly to the service of the sick poor.

Quite in accordance with modern theories of medicine, whereby one evil is utilised to conquer a greater, the surgeon of a large American railway read a paper recently on the beneficial results which sometimes follow shocks from railway accidents. Rheumatism, phthisis, gout, fever, ague, and epilepsy have all been dissipated after an experience of a railway accident, according to the instances given. Of course, it is well known that many varieties of nervous disease are curiously influenced by nervous shock; it must, however, be borne in mind that the essence of shock is the unexpected, and that, humanity apart, the curative influence of railway accidents, such as it may be, is, and must always remain, itself merely accidental.

New buildings and additions are progressing apace in the hospital world. At Dundee the Royal Infirmary is to have a new operating theatre, estimated at £1,500. At Edinburgh a new dental hospital has just been opened. The premises have cost about £6,000, and provide accommodation for 31 operators. In Ireland a cottage hospital at Little Bray is to be erected to the memory of the late Father Healy. The projected infirmary for County Tyrone is likely to be soon commenced, and plans for buildings for 60 patients are to be prepared. In America a new women's hospital is to be established at New York, and at the Montefiore Home for Chronic Invalids a new wing has been opened.

Dr. Archibald Garrod showed a case at the Dermatological Society which may possibly be put to wrong use by anti-vaccinationists. The patient was a boy three years of age. He was vaccinated when three months old. Up to that time his skin and body generally had appeared perfectly healthy, and he had come of a healthy family. After the vaccination an abundant eruption appeared upon the child's trunk. It consisted of ill-defined red spots, which changed first to a "lean-ham" colour and then to brown. The eruption has come and gone up to the present time, the child being now, as stated, three years old. People may presume that the vaccination was the cause of the eruption; and the members of the Dermatological Society did not question that such was the case. Nevertheless it is quite possible that the eruption, though *post hoc* was not *propter hoc*, as the following case will show. A few years ago a London medical man was sent for to vaccinate twins. On arriving he found that his supply of lymph was deficient, and he therefore vaccinated only one of the them. In the course of a few days he was hurriedly sent for, the messenger informing him that one of the twins was dead. He went with a sinking heart, being but a young practitioner, never doubting but that it was the vaccinated twin which had died. When he arrived he found that it was the unvaccinated. Had the fact been otherwise, might it not have been taken absolutely for granted that the vaccination was the cause of death? In like manner, may it not be the fact that the skin eruption in Dr. Garrod's patient is a coincidence rather than a consequence of the vaccination?

